# The rare multidrug cervical tubercular lymphadenitis in an infant from Iran: A case report and literature review

**DOI:** 10.1002/ccr3.3612

**Published:** 2020-12-04

**Authors:** Masoud Keikha, Fatemeh Askarizadeh, Mohammad Saeed Sasan, Hossein Joghatayee, Saman Soleimanpour

**Affiliations:** ^1^ Antimicrobial Resistance Research Center Bu‐Ali Research Institute Mashhad University of Medical Sciences Mashhad Iran; ^2^ Department of Microbiology and Virology Faculty of Medicine Mashhad University of Medical Sciences Mashhad Iran; ^3^ Tuberculosis Reference Laboratory Mashhad University of Medical Sciences Mashhad Iran; ^4^ Department of Pediatrics Faculty of Medicine Mashhad University of Medical Sciences Mashhad Iran

**Keywords:** cervical tuberculosis lymphadenitis, Iran, multidrug resistant, tuberculosis

## Abstract

Cervical‐TB lymphadenitis is the most frequent extrapulmonary manifestation of tuberculosis infection. There are limited documents (only five documents) on multidrug‐resistant cervical tubercular lymphadenitis, but there is no evidence for MDR‐cervical tuberculosis lymphadenitis in infants, which may occur in TB endemic regions.

## Graphical abstract

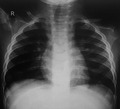

## 1 INTRODUCTION

Tuberculosis (TB) remains as a nightmare for the public health; TB has various forms, including the cervical tuberculosis lymphadenitis (CTL), classically known as “scrofula,” which is the most common form of extrapulmonary tuberculosis that often affects children. Spreading a strain of drug‐resistant tuberculosis (DR‐TB) is currently witnessed all over the world that is specifically observed in TB endemic countries. The present study demonstrates a report on a multidrug‐resistant cervical tuberculosis lymphadenitis (MDR‐CTL) case in a 2‐month age infant that was born from a mother with TB. Unfortunately, the infant did not respond to the first‐line antituberculosis therapy. Drug‐susceptibility testing was performed for the infant TB strains and the fingerprinting assay using the *IS6110*‐RFLP and spoligotyping was conducted to identify the potential source of infection. We found that the mother was infected with multiple strains (Delhi/CAS using *IS6110*‐RFLP and Beijing lineages by spoligotyping). Thus, it was possible for the infant to be infected congenitally. Moreover, the infant was initiated by ethionamide, moxifloxacin, amikacin, and linezolid based on the drug‐susceptibility pattern, and other members of the family also received moxifloxacin as a prophylactic MDR‐TB. Finally, the infant was fully recovered without recurrence of TB and his family was protected in that regard.

Tuberculosis is one of the most important infectious diseases that is considered among the top ten causes of mortality worldwide. Based on the report by the World Health Organization (WHO), about 10 million new cases were diagnosed with tuberculosis in 2019. In addition, there were 1.6 million related deaths in that year.^1^, ^2^ The rate of incidence of tuberculosis in Iran is about 22 per 100 000, and the mortality rate is 3.5 per 100 000. According to the reports, the rate of MDR‐TB resistance has been reported in Iran at about 1.3%‐5%.^3^, ^4^

While most of the clinical manifestations of tuberculosis are in the pulmonary basis, however, *Mtb* is capable of affecting all the organs, which is termed extrapulmonary TB (EPTB). In this respect, cervical tuberculous lymphadenitis is the most common form of EPTB, accounting for 25%‐30% of the cases.^5^ The incidence rate for the EPTB in Iran has also been reported to be approximately 2.5 cases per 100 000 people per year, which are mainly affecting children.^6^

Cervical tuberculosis lymphadenitis (CTL) typically involves the lymph nodes of the jugular, posterior triangle, and supraclavicular region, and the observed clinical manifestations of cervical tuberculosis lymphadenitis include fever, weight loss, rarely coughing, night sweat, chills, malaise, suppurative lymphadenitis, granulomatous inflammation, neck mass (1‐3 cm), fistula formation, and caseous necrosis.^7^, ^8^ However, cervical tuberculosis lymphadenitis can be misidentified with diseases such as malignancy, fungal infection, tularemia, actinomycosis, sarcoidosis, and nontuberculosis mycobacteria (NTM) lymphadenitis.^8^, ^9^

Based on the WHO recommendations, category III tuberculosis has been used since the beginning of 1997 to treat cervical tuberculosis and lymphadenitis cases.^8^ The development of drug resistance TB (particularly in TB endemic regions) has led to poor treatment outcomes in recent years.^10^ Thus, it is essential to introduce a new therapeutic regimen for the management and treatment of MDR cases of CTL patients.^8^, ^9^, ^11^, ^12^

There are limited reports on MDR‐CTL in the world; therefore, there is restricted information about patient management, diagnostic test, and treatment of MDR‐cervical lymphadenitis.^10^, ^13^, ^14^, ^15^, ^16^ Despite previous reports, herein we described the first report of cervical MDR‐tuberculosis lymphadenitis in a 2‐month‐old infant from Mashhad, Iran.

## 2 CASE PRESENTATION

In August 2018, a 32‐year‐old pregnant mother at her 34 weeks of gestation was referred to Dr Shariati Hospital in Mashhad, Iran, due to weight loss, fever, persistent, coughing, and dyspnea. She was diagnosed by sputum smear and culture examination to have pulmonary tuberculosis. The patient was treated for category I tuberculosis, and her symptoms became gradually negative in accordance with the sputum smear results. However, she delivered about one month later at 41 weeks’ gestation. Despite receiving prophylactic TB therapy (isoniazid), 55 days after his birth, the child (boy infant) was affected by fever (38.3°C), anorexia, and a painful swollen lesion in the neck area.

Since the mother had tuberculosis during pregnancy, the baby was also diagnosed as a “TB Case” and treated with isoniazid and rifampicin. Although the neck mass biopsy results showed evidence of inflammation, they were negative for the acid‐fast bacilli. The child's cervical swelling did not improve after about 4 months of antituberculosis therapy and had a pale colored discharge.

**Figure 1:**
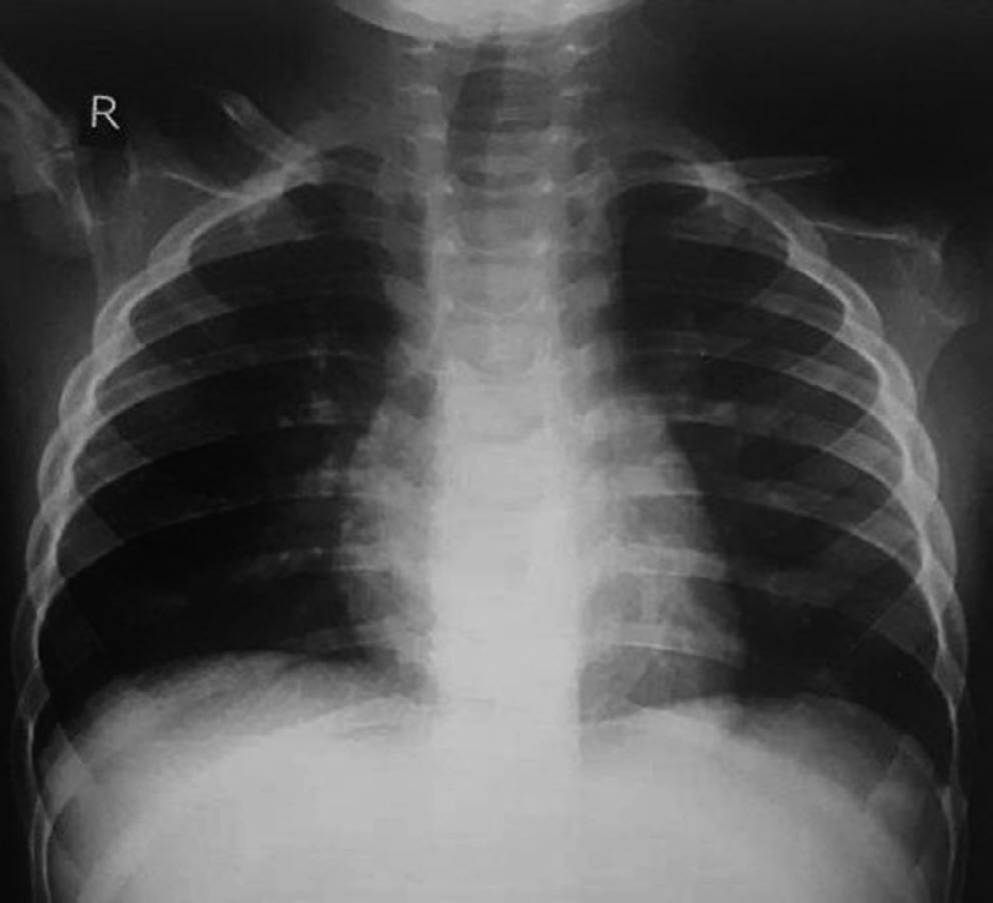
The CXR image of the patient revealed normal scene without remarkable sign except minor nodular and infiltrative lesion in the left lung

The patient was also negative for the HIV, HBV, and HCV tests. The patient had no evidence of organomegaly. CBC results included WBC: 24 000/mm^3^, RBC: 5200/μm, Hb: 11.5 g/dL, HCT: 37.4%, and platelets: 535 000/mm^3^. The serum levels of IgG, IgM, and IgA were 1500, 122, and 104 mg/dL, respectively. The parents of his patient were not satisfied about the lumbar puncture of the baby and the disseminated TB was ignored based on the radiological findings and negative culture of blood for Mtb. The sonography analyses of the internal organs such as liver, bile duct, pancreas, kidney, and bladder were normal and no evidence of pleural effusion; chest X‐ray (CXR) images of the patient's lung showed normal results (Figure 1).

The patient underwent FNA (fine‐needle aspiration) and the patient's biopsy specimen was sent to Tuberculosis Reference Laboratory in Mashhad for cytopathology and culture studies. Based on reported results from the neck mass biopsy, numerous evidences of multiple necrotizing granulomas with caseous lesions were observed; Ziehl–Neelsen staining results confirmed the existence of acid‐fast bacilli. The results of the “QuantiFERON assay” also identified the presence of an immunological response to *Mtb* infection.

The standard proportional method analysis was carried out according to the Clinical and Laboratory Standards Institute on Lowenstein‐Jensen medium, and resistance was observed to isoniazid, rifampicin, and ethambutol. According to the GeneXpert MTB‐RIF assay, the *Mtb* isolate was resistant to rifampicin. In addition, based on the drug molecular susceptibility test (DST) results performed by DNA sequencing, the considered isolate showed resistance to rifampicin, isoniazid, and ethambutol and was introduced as an MDR‐TB case. Due to the importance of identifying the source of infection, the *Mtb* isolates of the studied infant and her mother underwent genetic fingerprinting subjected to IS6110‐RFLP and spoligotyping. It was found that the infant isolate was from the Beijing lineage, whereas that of her mother was from the mixed infection with Beijing and Delhi/CAS lineages. Confirmation of IS6110‐RFLP results by Spoligotyping suggested that the mother had multiple strains as the consequence of recent transmission. It has been more probable that the patient was infected with *Mtb* as congenitally route. However, the patient was treated with a combination therapy including surgical drainage and antibiotic therapy by ethionamide, moxifloxacin, amikacin, and linezolid. Other members of the patient's family also received prophylactic MDR‐TB (with moxifloxacin). After one year and regular follow‐up of the patient, it was found that the patient was fully recovered, and no signs of reactivation were observed in the patient, his mother, and their other family members.

## 3 DISCUSSION

Cervical tuberculosis lymphadenitis is one of the most prevalent forms of extrapulmonary TB that often occurs in immunocompromised cases.^16^ Although Mtb often affects the lungs, in immune‐compromised cases, particularly children and HIV‐infected people, TB bacilli are spread through the lymphatic system due to the lack of an efficient and effective TB bacilli immune system, often occurring in the form of cervical tuberculosis lymphadenitis.^10^, ^16^, ^17^

Despite the fact that the CTL cases caused by DR‐TB are very limited, but the epidemiologic importance and diagnostic difficulties of managing and treatment of these cases are quite challenging because of the lack of specific guidelines for the treatment, particularly in the immunocompromised patients, who do not usually have granulomatous inflammation due to immune dysfunction. Moreover, PPD results in these patients are negative due to a weakened immune system.^10^, ^16^ Based on the available evidence, lung CXR is usually normal in cervical tuberculosis lymphadenitis patients, merely showing the abnormalities in 24%‐46% of these patients.^18^ Therefore, limited cases of cervical tuberculosis lymphadenitis caused by DR‐TB strains have been reported, most of which occur in the TB endemic countries (Table 1).

**Table 1:** The previous documents on CTL caused by DR‐TB strains with summary of the main clinical features

The authors	Age	Gender	Risk factors	Manifestations	Treatments	Clinical Outcomes	Ref
Ogundipe et al	22	Female	Contact with pulmonary TB	Slow growing right neck mass, positive TB quantiferon assay	Ethambutol, pyrazinamide, moxifloxacin, amikacin, linezolid, and cycloserine	Improved	12
Datta et al	32	Male	Not reported	Persistent glandular swelling, fever, reactive hyperplasia	Regimen based on the DST (24 mo)	Fully recovered	13
Mirsaeidi et al	19	Male	Immunocompetent	Lump in the right submandibular, mild anorexia and weight loss	Isoniazid, 500 mg daily, pyrazinamide 1500 mg daily, ofloxacin 600 mg daily, amikacin 500 mg daily, and clofazimine 200 mg daily	Improved`	14
Kant et al	28	Female	Immunocompetent	Bilateral cervical adenopathy with multiple discharging sinuses	Kanamycin (0.75 g every day), clofazimine (100 mg twice daily), clarithromycin (500 mg twice daily), cycloserine (750 mg everyday), ethionamide (750 mg every day), and levofloxacin (750 mg every day) for 6 mo	Fully recovered	15
Mittal et al	23	Female	Nursing student	Swelling in the right side of neck	Kanamycin 0.75 g, levoflox 750 mg, cycloserine 750 mg, ethionamide 750, PAS powder 10 gm and ethambutol 1.2 g, Pyrazinamide 1.5 g for 3 mo for case 1	improved	16
	35	Male	Previous CTL treatment with category I	Swelling in the left side of neck (relapse), loss of weight	Kanamycin 0.75 g, levoflox 750 mg, Cycloserine 750 mg, ethionamide 750, PAS powder 10 gm and ethambutol 1.2 g. for 6 mo	Fully recovered without recurrence	
	30	Male	Serum samples were positive for antibodies against HIV.	Enlarged right supraclavicular lymph node, lymph node showed chronic granulomatous inflammation	Antiretroviral treatment (lamivudine + stavudine +efavirenz) plus anti‐TB second‐line drugs for 6 mo	improved	

The present study was a rare report about cervical lymphadenitis caused by primary MDR‐TB strain in an infant that was born from a TB patient's mother. Fingerprinting of the infant and mother isolates showed that the mother was infected with multiple strains, whereas the child was infected with the strains of the Beijing genotype family. The MDR‐TB strain for the present case belonged to the Beijing genotype family, which was confirmed previously in the published Iranian reports. In a systematic review and meta‐analysis on the Iranian MDR‐TB cases, Tarashi et al (2017) found that the distribution of the Beijing genotype family is predominant among the MDR‐TB Iranian cases.^19^

The most common clinical manifestations and symptoms of cervical tuberculosis lymphadenitis include single or multiple painless lumps, lymphadenopathy, fistula formation (in some cases), weakness, low grade of fever, coughing, and pulmonary hilar lesion (if being involved and in case of primary lung infection) ^18^, ^19^, ^20^; the cervical tuberculosis lymphadenitis may lead to misdiagnosis in MDR‐TB cases of due to treatment failure or various patients manifestation such as negative PPD, lack of lung involvement, absence of granuloma formation, and coinfection with HIV or immune disorder.^21^ However, a review of the literatures has revealed that the mortality rate of MDR‐CTL cases is low, and there is not even a relapse in the untreated CTL cases (Table 1).

According to the literatures, CTL diagnosis is quite challenging. The sensitivity and specificity of the diagnostic methods for detection of cervical tuberculosis lymphadenitis are in a wide range. For example, the sensitivity of acid‐fast staining and culture methods for cervical tuberculosis lymphadenitis detection are estimated to be about 46%‐78% and 10%‐69%, respectively.^20^, ^22^ Moreover, culture also takes about 6‐8 weeks due to the slow growth nature of *Mtb* and is not quite appropriate, particularly in the MDR‐TB cases.^23^ Unfortunately, TB is possible to be misdiagnosed with other diseases due to treatment failure, particularly in TB endemic regions with higher number of MDR‐TB cases.^13^, ^14^, ^15^, ^16^ We have revealed that the fine‐needle aspiration is a reliable method for detection of cervical tubercular lymphadenitis. Gupta et al (1993) found that the sensitivity and specificity of FNA for diagnosis of cervical‐TB lymphadenitis were 88% and 96%, respectively.^24^

## 4 CONCLUSION

We reported the first case of MDR‐TB cervical lymphadenitis in a 2‐month‐old male infant worldwide. It was determined that his mother was infected by multiple *Mtb* strains, and possibly, the infant boy was infected as a congenital‐TB route. MDR‐CTL cases are more common in TB endemic regions. We provided a literature review and have revealed that MDR‐CTL is not usually fatal. Moreover, the fluoroquinolone is an appropriate therapeutic agent for the prophylactic aspect of contacts with the MDR‐TB cases.

## CONFLICT OF INTEREST

The authors declare that they have no competing interests.

## AUTHORS' CONTRIBUTIONS

MK and FA: writing original draft; MK and SS: writing—review and editing; MSS and HJ: investigation and data analysis; and MK and SS: supervision.

## ETHICAL APPROVAL

The ethical approval for performing this study was obtained from the Ethics Committee of the Mashhad University of Medical Sciences, and written informed consent was obtained from parent's patient.
